# Perceived strain of undergraduate medical students during a simulated first day of residency

**DOI:** 10.1186/s12909-018-1435-4

**Published:** 2018-12-29

**Authors:** Sophie Fürstenberg, Sarah Prediger, Martina Kadmon, Pascal O. Berberat, Sigrid Harendza

**Affiliations:** 1 Department of Internal Medicine, University Medical Center Hamburg-Eppendorf, Hamburg, Germany; 2 Faculty of Medicine, University of Augsburg, Deanery, Augsburg, Germany; 3 TUM Medical Education Center, School of Medicine, Technical University of Munich, Munich, Germany; 4 Universitätsklinikum Hamburg-Eppendorf, III. Medizinische Klinik, Martinistr. 52, D-20246 Hamburg, Germany

**Keywords:** 360-degree examination, Assessment, Consultation, Curriculum, Handover, Patient management, Residency, Strain, Undergraduate medical education

## Abstract

**Background:**

Residents face demanding situations on the job and have been found to perceive high levels of strain. Medical students also reported a high degree of strain and even depressive tendencies when entering their clinical rotations. The aim of this study was to explore the perceived strain of medical students from different undergraduate curricula and at different stages of academic advancement during different phases of an assessment simulating a resident’s first day in hospital.

**Methods:**

Sixty-seven undergraduate medical students participated in the following three phases of the assessment in the role of a resident: a consultation hour with five simulated patients, a management phase with interprofessional contact, and a patient handover with a colleague. They completed the Strain Perception Questionnaire (STRAIPER) after each phase. Students from different undergraduate curricula (VI: vertically integrated, *n* = 35 versus non-VI: not vertically integrated, *n* = 26) and different academic advancement (semester 10, *n* = 26 versus final year, *n* = 41) were compared.

**Results:**

All students showed the highest strain level after the management phase compared to the consultation hour and the handover. Medical students from a non-VI curriculum felt significantly more strain in the dimension of agitation (*p* < .05) after the consultation hour compared to students from a VI curriculum and compared to the management phase and the handover. No significant difference in perceived strain was found between students from semester 10 compared to final year students.

**Conclusions:**

During the consultation hour and the handover with a colleague medical students faced tasks which are familiar to them from undergraduate education. Their higher strain levels during the management phase might occur because they are confronted with unfamiliar tasks and decisions. Feeling responsible for the right actions in this phase of multitasking and professional interaction might have added to the strain students perceived during this phase. Patient management should be emphasized more in any type of undergraduate medical curriculum.

## Background

Handling busy clinical days while providing professional and compassionate patient care is an essential part of a resident’s work. Residents have been found to perceive high levels of strain and show abnormal burnout scores [1]. Feeling stressed during work can also negatively affect physician delivery of high-quality health care [2]. Even first year medical students already show higher levels of stress, anxiety, and depression than the general population facing the academic workload during undergraduate education [3, 4]. They also reported even greater stress and depressive feelings when entering their clinical rotations [5]. The experience of burnout symptoms can occur in medical students even already during the final year of undergraduate medical studies [6]. To support aspiring physicians already during their undergraduate training to cope with stress and burnout, a conceptual model has been proposed, which can help students to cultivate the skills to sustain their well-being throughout their careers while faced with an enormous workload and emotionally staining situations [7].

To prepare medical students well for clinical practice, competence-based curricula provide a seamless linkage from undergraduate education to postgraduate training [8]. Many medical schools worldwide are reshaping their undergraduate medical curricula to become competency-based [9–11]. The CanMEDS competency framework, designed for postgraduate medical education [12], served as a guideline for these developments. With medical curricula being developed towards competency-based education, assessment also needs to be adapted with respect to the constructive alignment theory [13]. In a pilot project, a competence-based assessment was developed which simulated the first day of a beginning resident [14]. The final year medical students encountered a consultation hour in the role of a beginning resident, similar to the physician-patient communication training they experience during undergraduate medical education [15]. After the consultation hour, they had to evaluate the cases and to establish diagnostic and treatment plans [16] while dealing with common disturbances occurring on a ward like interprofessional interactions with nursing staff and colleagues. Afterwards, they reported the management for each patient to a supervisor, which is a core activity in hospitals across all disciplines [17]. With this assessment, different extents of certain competencies, e.g. physician-patient communication, empathy, and patient management, could be detected in students from vertically integrated (VI) and non-integrated (non-VI) curricula [18]. Additionally, students from the VI curriculum ordered significantly less laboratory and radiology tests in this assessment with comparable diagnostic correctness compared with non-VI students [19]. Whether students experienced strain while performing the resident’s tasks is not known. Therefore, we developed a 360-degree assessment based on competencies regarded to be relevant for first year residents [20]. This assessment, where undergraduate medical students act in the role of residents included three phases (consultation hour, management phase, and handover to a colleague), an evaluation by supervisors, nursing staff, simulated patients, and residents, and a self-assessment with a special focus on feeling strained [21].

In this study, we focus on the strain perceived by medical students from different undergraduate curricula and with different academic advancement in their undergraduate studies during a simulated first day in hospital in the role of a beginning resident. We hypothesize that medical students from different undergraduate curricula differ in their perceived strain during the three different phases of the assessment. We also assume that students in their 10th semester differ in their perceived strain from final-year students.

## Methods

To determine strain perception of medical students from different undergraduate curricula, students participated in a newly designed assessment, which simulated the first workday of a resident [21]. The clinical environment was simulated and all interactions took place with real nurses, residents and attendings. The patients were played by actors. In the role of a resident, participants held a consultation hour with five simulated patients (one hour) followed by a management phase (two and a half hours), where they could organize the patients’ next diagnostic steps and interacted with other health care personnel. Eventually, they handed the patients over to a real resident (half an hour) [21].

After each of the three phases, students filled out the QCD (Short questionnaire on current disposition) by Müller and Basler [22], which we modified in the order and polarization of the items and renamed STRAIPER (Strain Perception Questionnaire). It includes the following six bi-polar items: tension (calm versus tense), doubt (confident versus doubtful), concern (unconcerned versus worried), agitation (unwound versus agitated), discomfort (comfortable versus uncomfortable), apprehensiveness (relaxed versus apprehensive). It serves the quantification of situation-dependent subjective mental strain. The scale includes values from 1 to 6, whereby 1 means a minimum and 6 a maximum of perceived strain in the respective item category. The internal reliability (Cronbach’s alpha) of the STRAIPER in our study sample is between .782 and .896.

Seventy students from three medical schools participated in the assessment. We excluded three students from the analysis who had not reached their 10th semester. Therefore, data from 67 students were analyzed: students from the University Medical Center Hamburg-Eppendorf (UKE, *n* = 35) and from the Carl von Ossietzky University of Oldenburg (*n* = 6) follow a vertically integrated (VI) undergraduate curriculum and students from the Technical University of Munich (TUM, *n* = 26) follow a non-VI undergraduate medical curriculum. The three different curricula all last six years. The non-VI curriculum in Munich includes two years of basic sciences training followed by a state examination and three years of clinical courses, followed by a state examination and a final practice year. The VI curriculum in Hamburg includes five years of integrated courses with a mix of basic sciences and clinical sciences from the beginning including elements of problem-based learning followed by a state examination and a final practice year. The VI curriculum in Oldenburg consists of five years training with integrated basic and clinical sciences including longitudinal clinical placements followed by a state examination and a final practice year. The assessment took place during three days in July 2017 at the University Medical Center Hamburg-Eppendorf (UKE). Students from semester 10, before the written part of the national licensing examination, and from the final year (semester 11 and 12) were invited by email and selected for participation on a first come first serve basis. Participating students were randomized with respect to the day and time (morning, afternoon) of participation and with respect to the raters (supervisors, nursing staff, and residents). They received a 25 Euro book voucher after completion of the assessment. Sociodemographic data were collected, i.e. age, gender, academic advancement (semester 10 or final year), university (Hamburg, Oldenburg, Munich), university place allocation (e.g. selection test), and the grade point average (GPA) of their high school degree. This study is part of a larger study [21] which was based on a pilot study [14] where strain was not assessed. The Ethics Committee of the Chamber of Physicians, Hamburg, confirmed the innocuousness of the study and its accordance with the Declaration of Helsinki. Participation was voluntary and anonymized and participants consented to their participation.

Means and standard deviations were calculated for the different groups of participants. Due to the fact that there was a significant difference in university place allocation between students from Hamburg and Munich – students from Munich were selected for medical school mostly by their high school degree and students from Hamburg were selected mostly by a test of basic sciences – the statistical analysis included a t-test and a two-way repeated analysis of covariance (MANCOVA) to study differences between these groups. Due to the low number of participants from Oldenburg (*n* = 6), they were excluded from the calculations comparing participants form the different universities. According to the authors of QCD, it captures a one-dimensional construct. However, our factor analysis showed at least two factors. Therefore, our analysis refers to the individual item level.

## Results

Of the 67 participants who were included in the analysis, 26 students had completed semester 10 and 41 students were in their final year. Table 1 shows the sociodemographic data of the participating students. The management phase was the most demanding phase for participants from semester 10 and the final year (Table 2). No significant difference in perceived strain was observed between students from semester 10 versus students in their final year with respect to any of the STRAIPER items and the three phases of the assessment.

**Table 1:** Gender and academic advancement of all participating students from Hamburg, Oldenburg, and Munich

	Hamburg (*n* = 35)		Oldenburg (*n* = 6)		Munich (*n* = 26)	
	*n*	%	*n*	%	*n*	%
Gender						
Male	18	51.4	3	50.0	17	65.4
Female	17	48.6	3	50.0	9	34.6
Academic advancement						
Semester 10	8	22.9	6	100.0	12	46.2
Final Year	27	77.1			14	53.8
University place allocation						
GPA of high school degree	9	25.7			19	73.1*
Selection test	17	48.6*	4	66.6	3	11.5
Other	9	15.7	2	33.4	3	11.5
Not specified					1	3.9

*GPA* grade point average; *p < .05 (between Hamburg and Munich)

**Table 2:** Level of strain perceived by students from semester 10 and final year during the 360-degree assessment

Perceived strain	Semester 10 (*n* = 26)			Final year (*n* = 41)		
	Consultation hour	Management phase	Handover	Consultation hour	Management phase	Handover
	*M ± SD*	*M ± SD*	*M ± SD*	*M ± SD*	*M ± SD*	*M ± SD*
calm (1) – tense (6)	3.58 ± 1.24	4.38 ± 1.24	3.04 ± 1.49	3.41 ± 1.43	4.41 ± 1.28	3.51 ± 1.42
confident (1) – doubtful (6)	3.23 ± 1.11	3.85 ± 1.12	3.12 ± 1.45	2.90 ± 1.22	3.27 ± 1.16	3.07 ± 1.21
unconcerned (1) – worried (6)	3.62 ± 1.10	4.08 ± 1.06	3.36 ± 1.25	3.29 ± 1.27	3.70 ± 1.22	3.29 ± 1.17
unwound (1) – agitated (6)	3.50 ± 1.11	4.08 ± 1.26	3.12 ± 1.42	3.32 ± 1.25	4.10 ± 1.17	3.05 ± 1.22
comfortable (1) – uncomfortable (6)	3.27 ± 1.00	3.85 ± 1.01	3.36 ± 1.08	3.17 ± 1.18	3.71 ± 0.98	3.12 ± 1.35
relaxed (1) – apprehensive (6)	3.35 ± 0.94	3.81 ± 0.80	3.00 ± 1.04	3.05 ± 1.14	3.54 ± 1.19	3.12 ± 1.19

**Fig. 1:**
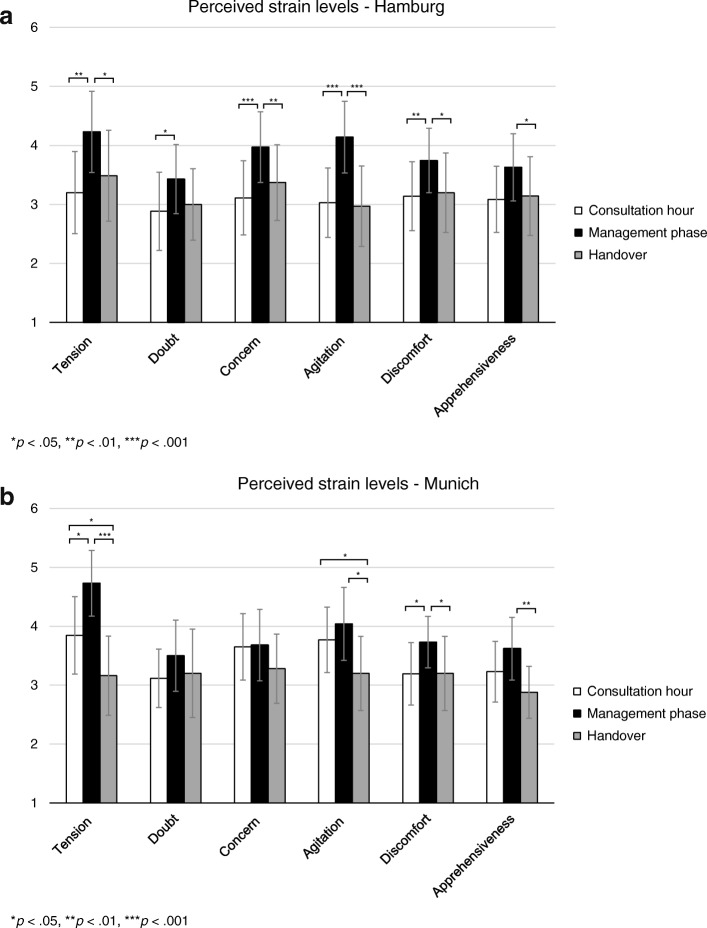
**a** Perceived strain during the three assessment phases by participating students from Hamburg (VI curriculum). **b** Perceived strain during the three assessment phases by participating students from Munich (non-VI curriculum)

Students from Hamburg (VI curriculum) and from Munich (non-VI curriculum) perceived the highest strain on every item level during the management phase compared to the consultation hour and the handover (Fig. 1a and b) with significant differences (*p* < .001 to *p* < .05) for all STRAIPER items except for doubt and concern. Students from Munich (Fig. 1b) felt significantly (*p* < .05) more tension (*M* = 3.85, *SD* = 1.31) and agitation (*M* = 3.77, *SD* = 1.10) during the consultation hour compared to the handover (tension: *M* = 3.16, *SD* = 1.34; agitation: *M* = 3.20, *SD* = 1.26). On the item level, students from Munich felt significantly more agitated (*M* = 3.77, *SD* = 1.11; *p* < .01, *d* = .64) during the consultation hour compared to students from Hamburg (*M* = 3.08, *SD* = 1.18).

The covariance analysis showed that the university place allocation, which is significantly different between Hamburg and Munich (Table 1), had no effect on students’ strain perception during any phase of the assessment. Neither the day nor the time of the day of the assessment, nor the respective evaluation by the different raters nor the athletic activity of the students had a significant effect on the students’ strain perception.

## Discussion

Residents and even medical students have been described to perceive high levels of strain [1, 3, 6]. In our 360-degree assessment simulating a busy first day of residency, medical students from a VI and from a non-VI curriculum felt the highest amount of strain in the role of a resident during the patient management phase. Several competencies related to management [23], i.e. prioritizing, interprofessional interaction, and handling of disturbances were required in this phase simultaneously. Taking a patient’s history and handing a patient over to a colleague might not cause as much strain because both are familiar tasks practiced during undergraduate medical education [15, 24]. Management, on the other hand, requires multitasking, which residents often learn on the job and not during undergraduate training. The more time physicians spent on multitasking the higher is the level of the strain they perceive [25]. Since multitasking can be necessary in many clinical situations and leads to higher failure rates, multitasking simulations might help undergraduate and postgraduate students to cope with such difficult situations [26]. Being familiar with multitasking situations after a simulation might also be helpful to reduce strain. Furthermore, feedback on performance, being challenged in unfamiliar contexts, and problem based learning might enhance the capability of managing several situations at the same time and to prioritize different tasks in medical education [27], which could lead to a reduction of strain felt in such situations. To reduce the strain medical students might have felt in the managing phase of our assessment do to interprofessional interaction, interprofessional experiences should be integrated further in undergraduate medical education [28]. The only significant difference we found between students from two universities with different types of curricula was a higher perception of agitation during the consultation hour by students from a non-VI curriculum (Munich) compared to students from a VI curriculum (Hamburg). This difference might be related to the fact that the assessment took place in Hamburg and the students from Munich had to familiarize themselves with the location of the assessment while the students from Hamburg were already familiar with the rooms from their OSCEs.

Interestingly, there was no significant difference in perceived strain between students from semester 10 and from the final year and students from both groups felt the highest strain levels after the management phase. Apparently, taking a history and handing a patient over to a colleague are competencies which are acquired and tested during the entire undergraduate medical education in a standardized way [29], which might help to reduce strain while performing theses task. Usually, tasks in OSCEs have to be performed successively and students are never challenged to prioritize patients or tasks, which is an important competence in the daily routine of a resident’s work in emergency departments and on the ward [30]. Being faced with such a situation during the patient management phase of our assessment but not having been able to practice this competence neither up to semester 10 nor during the final year might explain why strain perception was highest for both groups and why we found no difference between the two groups. As one perceived stressor or stress provoking factor when entering the final year, medical students described timing and organizational issues [31]. This might be an additional clue why the management phase of our assessment provoked the highest strain levels, which were not significantly different between students from semester 10 and the final year, for the management phase. Furthermore, no specific teaching focus seems to be on patient management during the final year, where it should have been a learning objective, as many students complain about being busied with routine tasks [32]. Furthermore, when final year students participated in a project with video-based on-ward supervision, none of the participants chose patient management tasks while history-taking was chosen most frequently [33]. Therefore, it might be useful to implement patient management tasks including prioritizing and interprofessional interaction during the entire curriculum to reduce medical students strain when they enter the final year. Furthermore, introducing entrustable professional activities into the undergraduate curriculum [34] including simulations for management on a ward [35] might also lead to a reduction of perceived strain in medical students while performing such tasks.

A weakness of our study is that students were chosen to participate in this assessment on a first come, first serve basis. Hence, only very motivated students might have participated. Therefore, the actual strain students feel during the different phases of our assessment might differ, if all students of the same semester are included. The sociodemographic data of our participating students show a significant difference in university place allocation between students form Hamburg and Munich which is another weakness. However, differences between students admitted to university by different parameters of selection diminish during the progress of undergraduate education [36]. The medical school at the University of Oldenburg is much smaller than the medical faculties at the TU Munich and at the University of Hamburg, which resulted in a much lower number of voluntary participants form Oldenburg in our study. Due to the low number of participants from Oldenburg, this medical school, which also offers a VI curriculum, could not be included in the comparison between students from different curricula. However, participants could be included in the comparison of students from semester 10 versus students in their final year. Despite these limitations, the management phase could be identified as the most strainful part of the assessment. Hence, patient management during multitasking situations might be special learning objective during undergraduate medical education, which reflects the work situation of residents.

## Conclusion

Students from different undergraduate curricula show the highest strain levels after the management phase of an assessment simulating the day of a beginning resident. The consultation hour and the handover might be causing less strain because students are more familiar with these tasks from their undergraduate studies. Management in multitasking situations including prioritizing tasks and acting in an interprofessional team seem to be competencies which require a greater focus during undergraduate medical training to reduce strain when confronted with unfamiliar tasks and decisions. This study was a test of this type of assessment and its effects on students’ feeling of strain. Since it has been successfully implemented, larger studies will follow.

## Abbreviations

ANCOVA: Analysis of covariance
EPA: Entrustable professional activities
GPA: Grade point average
Non-VI: Not vertically-integrated curriculum
OSCE: Objective structured clinical examination
STRAIPER: Strain perception questionnaire
TUM: Technical University of Munich
UKE: University Medical Center Hamburg Eppendorf
VI: Vertically-integrated curriculum
